# A network approach to the symptom-level associations between smoking and posttraumatic stress disorder (PTSD) among young adults exposed to childhood sexual abuse

**DOI:** 10.7189/jogh.13.04037

**Published:** 2023-06-23

**Authors:** Yu Jin, Shicun Xu, Xianyu Luo, Yinzhe Wang, Jiaqi Li, Beixiang Liang, Hui Li, Xiaofeng Wang, Xi Sun, Yuanyuan Wang

**Affiliations:** 1 College of Education for the Future, Beijing Normal University, Beijing, China; 2 Northeast Asian Research Center, Jilin University, Changchun, China; 3 Department of Population, Resources, and Environment, Northeast Asian Studies College, Jilin University, Changchun, China; 4 China Center for Aging Studies and Social-Economic Development, Jilin University, Changchun, China; 5 Vanke School of Public Health, Tsinghua University, Beijing, China; 6 Key Laboratory of Brain, Cognition and Education Sciences, Ministry of Education, China, South China Normal University, Guangzhou, China; 7 School of Psychology, Center for Studies of Psychological Application, and Guangdong Key Laboratory of Mental Health and Cognitive Science, South China Normal University, Guangzhou, China; 8 School of Public Health, Jilin University, Changchun, China

## Abstract

**Background:**

Previous empirical literature has examined the associations between childhood sexual abuse (CSA) exposure, posttraumatic stress disorder (PTSD), and smoking. However, few studies examined symptom-level associations between smoking and PTSD among CSA victims. Thus, the aims of this study were 1) to explore symptom-level associations between smoking and PTSD among combustible cigarette (CC) and electronic cigarette (EC) users exposed to CSA and 2) to compare the differences manifested in two network structures between EC and CC users with CSA experiences.

**Methods:**

This cross-sectional study covers all 63 universities and colleges in Jilin province, China, from October 26 to November 18, 2021. A total of 117 769 students participated in this study, while 3479 young adults were exposed to CSA (3.62%, 95% CI = 3.50%-3.73%). Childhood sexual abuse, PTSD, and smoking symptoms were measured using the Childhood Trauma Questionnaire-Short Form (CTQ-SF), 10-item Trauma Screening Questionnaire (TSQ-10), and the 6-item Fagerstrom Test for Nicotine Dependence (FTND-6), respectively. In addition, network analysis was applied to analyse psychopathological symptoms between EC and CC users with CSA experiences. Both the edges and centralities were computed, and the network properties were compared among the two groups.

**Results:**

Four symptoms of PTSD (i.e. emotional cue reactivity, hypervigilance, nightmares, and difficulty concentrating) were both central and bridge symptoms between PTSD and smoking among EC and CC users with CSA experiences. Moreover, compared with CC users with CSA, there were significantly stronger associations between “nightmares” – “difficulty with restrictions” and “irritability / anger” – “more during wake up” among young EC users with CSA.

**Conclusions:**

The four symptoms (i.e. emotional cue reactivity, hypervigilance, nightmares, and difficulty concentrating) were keystones for treatments or interventions targeting these CSA victims with PTSD and smoking symptoms. Increasing efforts should be taken to restrict morning smoking among EC users with CSA. In addition, target interventions and strategies founded on these core symptoms and associations should be implemented to relieve the comorbid PTSD and smoking in EC and CC users with CSA experiences.

Childhood sexual abuse (CSA) is a common type of childhood trauma [1], which is a global phenomenon that affects millions of children [2]. A comprehensive review covering 2000 to 2017 showed that the median prevalence of CSA among girls in Europe, Australia, and North America was 14%, 29%, and 20%, respectively [3]. Furthermore, exposure to CSA is associated with various mental disorders, such as depression, anxiety, posttraumatic stress disorder (PTSD), and substance use [4,5], which might have downstream consequences on individuals’ quality of life [6].

The previous empirical literature has examined the associations between exposure to CSA, cigarette smoking, and PTSD. PTSD is one of the serious mental health outcomes among individuals with CSA experiences. These symptoms, such as intrusive thoughts, nightmares, sleep disturbance, and hypervigilance, often last long after the CSA experiences [7,8]. Furthermore, it has been well documented that people with experiences of CSA have been prone to perform adverse behaviours like smoking [9] compared to the general population. For example, in a large national sample of adolescents, CSA experiences would increase the risk of smoking [10]. Another study reported that trauma-exposed college students with PTSD showed increased cigarette smoking compared to those without trauma-exposed histories [11]. Additionally, findings from several studies underscored a strong association between PTSD and smoking [12] and that PTSD had been documented as a well-defined predictor of smoking relapse [13]. According to the theoretical smoking models, CSA victims with PTSD would like to smoke to modulate general negative affect [14,15]. For trauma-exposed smokers, the belief in reducing the negative effect mediates the relation between PTSD severity and cigarette smoking [16]. Therefore, the comorbidity of PTSD and smoking is prevalent among young adults with CSA experiences.

With the development of electronic cigarettes (EC), the prevalence of using EC has dramatically risen among young adults over the past decade globally [17]. EC delivers a nicotine-containing aerosol to users by heating a solution typically made up of propylene glycol or glycerol (glycerin), nicotine, and flavoured agents [18]. A national survey reported that the current use of EC increased from 1.5% in 2011 to 20.8% in 2018, although the use of combustible cigarettes (CC) decreased among high school students from the United States [19,20]. Compared with traditional CC, young adults’ rising interest in EC could be attributed to its aggressive marketing, alluring tastes, beliefs of lower harm, social media influences, and covertly used designs [21-24]. However, considering the differences between EC and CC, such as appearance, operating principle, and the level of plasma nicotine [17,18], the associations of symptoms between PTSD and smoking would be different among EC users and CC users exposed to CSA. Thus, it becomes essential to investigate symptom-level associations between smoking and PTSD among EC and CC users exposed to CSA.

Network analysis is a methodology to explore the associations of symptoms between different disorders [25,26]. Nodes and edges are included in psychological network structures. The network nodes can represent psychological characteristic variables, such as symptoms and behaviours, while the network edges represent statistical relationships, such as biased correlations and predictive relationships between variables. The thicker and deeper-colour edges of the two nodes refer to closer influential relationships [27]. The central symptom refers to nodes with the highest centrality and influential strength, which could be identified by the expected influence (EI) index [28]. Meanwhile, bridge symptoms, which are connected and influenced by each other, show co-occurring psychiatric symptoms within networks, which could be estimated by bridge expected influence (BEI) indices [29]. Such an approach emphasizes that the activation of a node in each psychological structure will likely simultaneously activate a node in another. This conceptualization has been implemented in models covering a range of psychiatric disorders, including PTSD [30,31]. This approach provides a new framework for conceptualizing the associations between PTSD and smoking.

This study used network analysis to examine the associations between PTSD and smoking symptoms in EC and CC users exposed to CSA. Moreover, these results have significant implications for future interventions targeting smoking cessation and alleviating PTSD symptoms. The objectives of this study were 1) to explore symptom-level associations between smoking and PTSD among EC and CC users exposed to CSA and 2) to compare the differences of two network structures between EC and CC users with CSA experiences.

## METHODS

### Study design and settings

This cross-sectional study, following the Strengthening the Reporting of Observational Studies in Epidemiology (STROBE) guidelines [32], covers all 63 universities and colleges in Jilin province, China, from October 26 to November 18, 2021. All colleges in Jilin province forwarded their students a Quick Response code (QR Code) to complete an electronic questionnaire. The inclusion criteria included: 1) aged 16 years or older; 2) students studying in universities or colleges in Jilin province, China; 3) able to understand Chinese and the assessment content.

Methods were carried out following the 1964 Helsinki Declaration and its amendments in 2013 and with ethical standards. Jilin University granted ethical approval for this study. All participants provided electronic informed consent.

### Measurements

#### Childhood sexual abuse

The experience of childhood sexual abuse (CSA) was measured by the Childhood Trauma Questionnaire-Short Form (CTQ-SF) [33], which contains 28 items, a self-report inventory developed to measure five types of abuse or neglect in childhood or adolescence. The Likert-type answer format ranges from “1” (never true) to “5” (very often true). Considering summing up scores from five-type subscales, the total scores vary from 5 to 25. According to the CTQ-SF subscale scores, a cutoff score of> = 8 could define the experience of CSA [34]. It shows good reliability and validity among Chinese undergraduates, with a Cronbach’s alpha coefficient of 0.77 [35].

### PTSD symptoms

The 10-item Trauma Screening Questionnaire (TSQ-10) [36], adapted from the PTSD Symptom Scale [37], is a self-report scale to measure traumatic events. The items were designed to detect symptoms of trauma exhibited by participants at least twice in the past week. Respondents were asked to answer the question of whether they had experienced these items using “Yes” (scored 1) or “No” (scored 0), and six or more positive responses indicated that the respondent was at risk of PTSD. The total minimum score is zero, and the maximum is ten. A broad spectrum of studies examined that the TSQ-10 displayed good validity and reliability in different countries [38-40]. It shows good internal consistency at a Cronbach’s alpha coefficient of 0.93, with a good sensitivity of 0.93 and specificity of 0.63 in Chinese university students [41]. This study also examined the good internal consistency of 0.85 among university students in the Chinese mainland.

#### Smoking symptoms

The 6-item Fagerstrom Test for Nicotine Dependence (FTND-6) was used in various countries to measure cigarette dependence [42]. The six items of FTND-6 include 1) “time to smoke the first cigarette”, 2) “difficulty with restrictions”, 3) “smoking desire”, 4) “cigarettes amount”, 5) “more during wake up”, and 6) “smoking when ill”. Of the six items on the FTND-6, three Yes / No items scored 1 (yes) and 0 (no). The other three items were scored from 0 to 3. Summing all items to obtain the total scores, ranging from 0 to 10, was used to detect the smoking level of smoking [43]. The FTND-6 could be an outcome assessment tool with sensitivity, reliability, and validity for the research of smoking symptoms [44]. In the Chinese population, the FTND-6 has also achieved internal consistency [45,46].

### Statistical analysis

#### Descriptive analysis

This study covers 117 749 university and college students. Excluding missing data, 96 218 participants remained, among which a subset of 3479 with CSA experiences was analysed. All students who experienced CSA were arranged into two groups according to their cigarette consumption types (combustible or electronic). Sociodemographic variables included participants’ age, sex, residence, ethnicity, family type, current annual income, and whether they were only-child status. Age and scaled scores (ie, CTQ-SF, TSQ-10, FTND-6) were continuous variables, and all the other sociodemographic variables were divided into two or more categories.

There were two steps to processing missing data. At first, the missing data of less than 5% of the total sample of sociodemographic variables were deleted. Then, replaced the missing data of each scoring scale with its intermediate value [47]. After dealing with the missing data, all the efficient data were analysed combined with R programming software and the IBM SPSS version 26.0, of which categorical variables were compared by χ^2^ analysis and continuous variables by the independent *t* test to investigate the differences in the CC and EC groups.

#### Network estimation

Partial correlation network analyses were used to assess the association between PTSD and smoking symptoms among participants with CSA by R package “qgraph” [48,49]. For each node, expected influence (EI) represents the summed weight of all its positive and negative edges with its immediate neighbouring nodes in the network [50]. The bridge EI (bEI) measures the role of a symptom as a linking device between PTSD and smoking behaviours. Our investigations also determined whether differences existed in network characteristics between EC and CC users with CSA experiences with the R package “NetworkComparisonTest” version 2.0.1 [51]. Computing confidence intervals assessed the network’s accuracy and stability (CIs), and the correlation stability coefficient (CS-C) bootstrapped difference tests [48,52-54].

## RESULTS

### Descriptive statistics

A total of 117 749 students were invited to participate, among which 96 218 satisfied the study inclusion criteria and completed the questionnaire. Among the 96 218 participants, 3479 young adults have CSA experiences, contributing to a prevalence of 3.62% (95% CI = 3.50%-3.73%). Among these CSA victims (ie, 675 CC users and 286 EC users), the prevalence of PTSD (total score of TSQ-10 > 5) was 47.02% (95% CI = 45.36%-48.70%), the prevalence of nicotine dependence (total score of FTND-6 > 5) was 5.66% (95% CI = 4.92%-6.48%). The mean (standard deviation (SD)) value of the total score of PTSD among CSA victims was 4.32 (3.14). **Table 1** shows the sociodemographic characteristics of participants, and **Table 2** presents the basic information on each scale and their descriptive item statistics.

**Table 1:** Sociodemographic characteristics of participants who experienced CSA, CC and EC groups

	Sexual abuse (n = 3479)	Combustible cigarettes (n = 675)	Electronic cigarettes (n = 286)	*χ^2^ / T*	*P* value
Sex					
*Male*	1699 (48.8)	574 (85.0)	224 (78.3)	6.43	<0.05
*Female*	1780 (51.2)	101 (15.0)	62 (21.7)		
Residence					
*City*	1788 (51.4)	358 (53.0)	166 (58.0)	2.03	0.154
*Town and county*	1691 (48.6)	317 (47.0)	120 (42.0)		
Ethnic					
*Han*	3119 (89.7)	600 (88.9)	265 (92.7)	3.17	0.075
*Others*	360 (10.3)	75 (11.1)	21 (7.3)		
Family type					
*Nuclear family*	2272 (65.3)	425 (63.0)	191 (66.8)	2.35	0.309
*More than three generation*	651 (18.7)	137 (20.3)	46 (16.6)		
*Others*	556 (16.0)	113 (16.7)	49 (17.1)		
Current annual income					
<US$940*	1052 (30.2)	203 (30.1)	83 (29.0)	0.443	0.931
*US$940-2199*	1047 (30.1)	169 (25.0)	68 (23.8)		
*US$2200-3599*	553 (15.9)	107 (15.9)	48 (16.8)		
*≥US$3600*	827 (23.8)	196 (29.0)	87 (30.4)		
Only-child					
*Yes*	1698 (48.8)	397 (58.8)	183 (64.0)	2.25	0.134
*No*	1781 (51.2)	278 (41.2)	103 (36.0)		
				*T*	
Age, years, mean (SD)	19.60 (1.71)	19.60 (1.72)	19.58 (1.69)	0.145	0.885
*FTND-6*	2.20 (2.26)	2.26 (2.23)	2.07 (2.32)	1.158	0.247
*PTSD-10*†	4.63 (3.22)	4.76 (3.31)	4.76 (3.27)	-0.10	0.992

CC – combustible cigarettes, EC – electronic cigarettes, FTND-6 – Fagerstrom Test for Nicotine Dependence-6, PTSD-10 – Posttraumatic Stress Disorder-10

*US dollars (US$) = 7.18 renminbi (RMB).

†Measured by the 10-item Trauma Screening Questionnaire.

**Table 2:** Basic information on scales and descriptive item statistics

	Symptoms	Items	Mean (SD)		Predictability	
**CC**	**EC**	**CC**	**EC**
FTND-6	Time to smoke the cigarette	1. How soon after you wake up do you smoke your first cigarette?	1.04 (1.12)	0.78 (1.13)	31%	27%
	Difficulty with restrictions	2. Do you find it difficult to refrain from smoking in places where it is forbidden, eg, in church, at the library, cinema, etc.?	0.15 (0.36)	0.24 (0.43)	13%	22%
	Smoking desire	3. Which cigarette would you hate most to give up?	0.29 (0.46)	0.19 (0.39)	23%	26%
	Cigarettes amount	4. How many cigarettes / day do you smoke?	0.35 (0.61)	0.44 (0.89)	24%	10%
	More during wake up	5. Do you smoke more frequently during the first hours of waking than during the rest of the day?	0.15 (0.36)	0.19 (0.39)	21%	22%
	Smoking when ill	6. Do you smoke if you are so ill that you are in bed most of the day?	0.28 (0.45)	0.23 (0.42)	22%	24%
PTSD-10*	Intrusive thoughts	1. Upsetting thoughts or memories about the event that have come into your mind against your will	0.61 (0.49)	0.60 (0.49)	33%	32%
	Nightmares	2. Upsetting dreams about the event	0.49 (0.50)	0.49 (0.50)	40%	44%
	Flashbacks	3. Acting or feeling as though the event were happening again	0.64 (0.48)	0.64 (0.48)	33%	33%
	Emotional cue reactivity	4. Feeling upset by reminders of the event	0.55 (0.50)	0.57 (0.50)	45%	42%
	Physiological cue reactivity	5. Bodily reactions (such as fast heartbeat, stomach churning, sweatiness, dizziness) when reminded of the event	0.45 (0.50)	0.45 (0.50)	38%	38%
	Sleep disturbance	6. Difficulty falling or staying asleep	0.43 (0.49)	0.42 (0.49)	34%	33%
	Irritability / anger	7. Irritability or outbursts of anger	0.37 (0.48)	0.38 (0.49)	39%	42%
	Difficulty concentrating	8. Difficulty concentrating	0.49 (0.50)	0.51 (0.50)	41%	42%
	Hypervigilance	9. Heightened awareness of potential dangers to yourself and others	0.35 (0.48)	0.32 (0.47)	46%	46%
	Exaggerated startle response	10. Feeling jumpy or being startled at something unexpected	0.38 (0.48)	0.38 (0.49)	41%	39%

SD – standard deviation, CC – combustible cigarettes, EC – electronic cigarettes, FTND-6 – Fagerstrom Test for Nicotine Dependence-6, PTSD-10 – Posttraumatic Stress Disorder-10

*Measured by the 10-item Trauma Screening Questionnaire.

### Network structure

**Figure 1:**
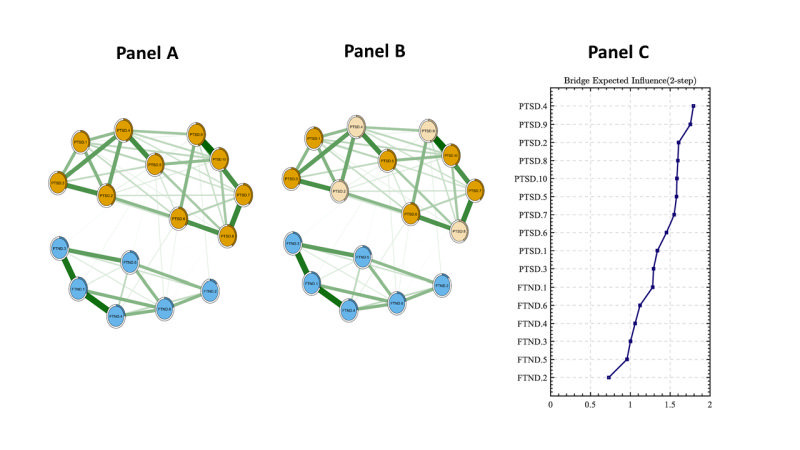
The global network and bridge network structures of PTSD and smoking symptoms among CC users exposed to CSA (n = 675). **Panel A.** Network structure, the symptomatic relationships between PTSD and smoking symptoms. **Panel B.** Bridge symptoms, the nodes of PTSD community connecting to smoking symptoms. **Panel C.** bEI values, the bridge expected influence index to identify the bridge symptoms. Notes: PTSD.1 – intrusive thoughts, PTSD.2 – nightmares, PTSD.3 – flashbacks, PTSD.4 – emotional cue reactivity, PTSD.5 – physiological cue reactivity, PTSD.6 – sleep disturbance, PTSD.7 – irritability / anger, PTSD.8 – difficulty concentrating, PTSD.9 – hypervigilance, PTSD.10 – exaggerated startle response, FTND.1 – time to smoke the cigarette, FTND.2 – difficulty with restrictions, FTND.3 – smoking desire, FTND.4, – cigarettes amount, FTND.5 – more during wake up, FTND.6 – smoking when ill. CC – combustible cigarettes, FTND – Fagerstrom Test for Nicotine Dependence, PTSD – Posttraumatic Stress Disorder

As shown in **Figure 1**, panel A and **Figure 1**, panel B, the global network and the bridge network structures of PTSD and smoking symptoms among CC users exposed to CSA were presented (n = 675).

The average predictability of nodes was 0.328, meaning 32.8% of the variance can be explained by its neighbouring nodes. In this network model, node PTSD.4 (“emotional cue reactivity”) had the highest EI index, followed by node PTSD.9 (“hypervigilance”), PTSD.2 (“nightmares”), and PTSD.8 (“difficulty concentrating”). Furthermore, these four symptoms were also bridge symptoms between PTSD and smoking. These results indicated that these four symptoms were the most crucial and meaningful for understanding the symptoms’ associations between PTSD and smoking among CC users with CSA (**Figure 1**, panel C). Additionally, the associations between PTSD and smoking have also been detected, such as PTSD.6 (“sleep disturbance”) – FTND.6 (“smoking when ill”), PTSD.5 (“physiological cue reactivity”) – FTND.1 (“time to smoke the first cigarette”), PTSD.9 (“hypervigilance”) – FTND.3 (“smoking desire”). Figure S1 in the **Online Supplementary Document** displays the symptom correlations between PTSD and FTND.

**Figure 2:**
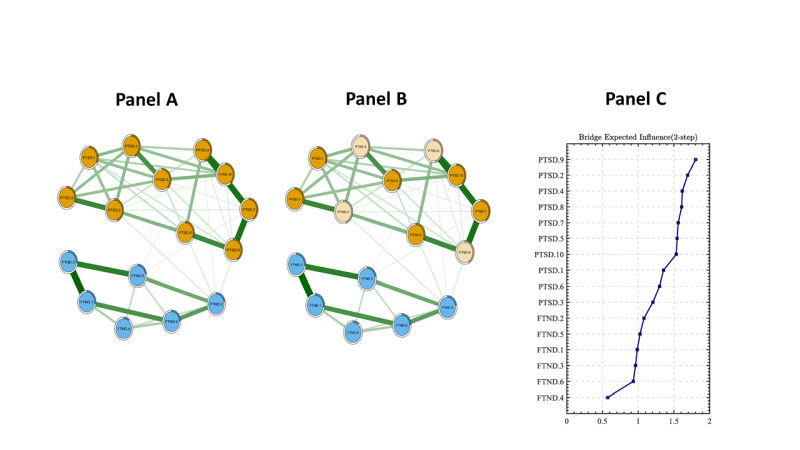
The global network and bridge network structures of PTSD and smoking symptoms among EC users exposed to CSA (n = 286). **Panel A.** Network structure, the symptomatic relationships between PTSD and smoking symptoms. **Panel B.** Bridge symptoms, the nodes of PTSD community connecting to smoking symptoms. **Panel C.** bEI values, the bridge expected influence index to identify the bridge symptoms. Notes: PTSD.1 – intrusive thoughts, PTSD.2 – nightmares, PTSD.3 – flashbacks, PTSD.4 – emotional cue reactivity, PTSD.5 – physiological cue reactivity, PTSD.6 – sleep disturbance, PTSD.7 – irritability / anger, PTSD.8 – difficulty concentrating, PTSD.9 – hypervigilance, PTSD.10 – exaggerated startle response, FTND.1 – time to smoke the cigarette, FTND.2 – difficulty with restrictions, FTND.3 – smoking desire, FTND.4 – cigarettes amount, FTND.5 – more during wake up, FTND.6 – smoking when ill. EC – electronic cigarettes, FTND – Fagerstrom Test for Nicotine Dependence, PTSD – Posttraumatic Stress Disorder

As shown in **Figure 2**, panel A and **Figure 2**, panel B, the global network and the bridge network structures of PTSD and smoking symptoms among EC users exposed to CSA were presented (n = 286).

The average predictability of nodes was 0.326, meaning 32.6% of the variance can be explained by its neighbouring nodes. In this network model, PTSD.9 (“hypervigilance”), PTSD.2 (“nightmares”) and PTSD.8 (“difficulty concentrating”), and PTSD.4 (“emotional cue reactivity”) were both central symptoms and bridge symptoms (**Figure 2**, panel C). In addition, the associations between PTSD and smoking also have been detected, such as PTSD.2 (“nightmares”) – FTND.2 (“difficulty with restrictions”), PTSD.9 (“hypervigilance”) – FTND.2 (“difficulty with restrictions”) and PTSD.9 (“hypervigilance”) – FTND.5 (“more during wake up”).

### Network comparison

**Figure 3:**
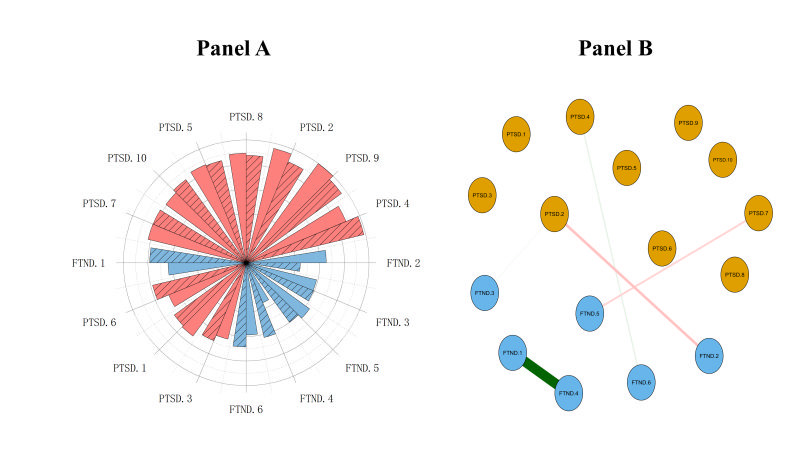
Network comparison between EC and CC groups. **Panel A.** Comparison of EI values of each node among EC and CC network groups. The column with solid lines combined into the CC group, and the column without solid lines combined into the EC group. **Panel B.** Comparison of correlations of each node among EC and CC groups. Green lines reflect the stronger correlations in CC group, while red lines reflect the stronger correlations in EC group. Notes: PTSD.1 – intrusive thoughts, PTSD.2 – nightmares, PTSD.3 – flashbacks, PTSD.4 – emotional cue reactivity, PTSD.5 – physiological cue reactivity, PTSD.6 – sleep disturbance, PTSD.7 – irritability / anger, PTSD.8 – difficulty concentrating, PTSD.9 – hypervigilance, PTSD.10 – exaggerated startle response, FTND.1 – time to smoke the cigarette, FTND.2 – difficulty with restrictions, FTND.3 – smoking desire, FTND.4 – cigarettes amount, FTND.5 – more during wake up, FTND.6 – smoking when ill. CC – combustible cigarettes, EC – electronic cigarettes, FTND – Fagerstrom Test for Nicotine Dependence, PTSD - Posttraumatic Stress Disorder

When comparing the EI value of each node, we found that EI values followed the following ranks of PTSD.4>PTSD.9>PTSD.2>PTSD.8 in the CC network and PTSD.9>PTSD.2>PTSD.8>PTSD.4 in the EC network. In other words, PTSD.4 (“emotional cue reactivity”) was the most central symptom in the network model among CC users. At the same time, it was the fourth important symptom in EC users’ network model (**Figure 3**, panel A).

When comparing other correlations in two network models, there were significantly stronger correlations in FTND.1 (“time to smoke the first cigarette”) – FTND.4 (“cigarettes amount”), PTSD.4 (“emotional cue reactivity”) – FTND.6 (“smoking when ill”), and PTSD.2 (“nightmares”) – FTND.3 (“smoking desire”) in the network model of PTSD and smoking symptoms among CC users with CSA (*P* < 0.05). Meanwhile, there were significantly stronger correlations in PTSD.2 (“nightmares”) – FTND.2 (“difficulty with restrictions”) and PTSD.7 (“irritability / anger”) – FTND.5 (“more during wake up”) in the network model of PTSD and smoking symptoms among EC users with CSA (*P* < 0.05) (**Figure 3**, panel B).

### Network stability

First, the network stability was measured by computing correlations between the node EI index of the original sample and gradually decreasing bootstrapped subsamples (Figure S2 in the **Online Supplementary Document**). EI index showed stability in both network structures (CS-C = 0.75 in CC; CS-C = 0.67 in EC). Second, the confidence intervals of edge weights were estimated through non-parametric CIs. The results revealed that the precision of edges was acceptable in these two network models (Figure S3 in the **Online Supplementary Document**). Third, the results of the bootstrapped difference tests also showed that several comparisons between edge weights were statistically significant in both network models (Figure S4 in the **Online Supplementary Document**).

## DISCUSSION

In this large-scale and cross-sectional study, the symptomatic associations between cigarette consumption and PTSD among young adults experiencing CSA were investigated. Four symptoms of PTSD (i.e. emotional cue reactivity, hypervigilance, nightmares, and difficulty concentrating) were both central and bridge symptoms for PTSD and smoking symptoms in the network model among young adults with CSA experiences. These results indicated that these four symptoms were crucial for treatment or intervention targeting CSA victims with PTSD and smoking behaviours. In specific, there were significant association differences between CC and EC users with CSA experiences, in terms of significantly stronger associations between “time to smoke the first cigarette” – “cigarettes amount”, “emotional cue reactivity” – “smoking when ill” among CC users with CSA, and significantly stronger associations between “nightmares” – “difficulty with restrictions” and “irritability / anger” – “more during wake up” among EC users with CSA.

The reporting rate of CSA in this study was lower than it in previous studies, estimating a prevalence of 3.62% (95% CI = 3.50%-3.73%) in Chinese university students. Previous studies reported, in China, the overall prevalence of CSA ranges from 8.9% to 27.5% [55-57]. A comprehensive review covering 2000 to 2017 showed that the median prevalences of CSA in Europe, Australia, and North America were 14%, 29%, and 20%, respectively [3]. This great discrepancy may be attributed to the population of study participants, samples from different regional or ethnic backgrounds, or various data-collecting methodologies. At first, participants in this study were mostly from the Han ethical nuclear family, in which adults would often follow the conventional Confucian moral rules and strong self-discipline about behavioural boundaries, aiming to create a harmonious family atmosphere [58], thus may decrease the risk of adverse abuse behaviours in their children. Meanwhile, compared to previous research based on the clinical sample, the results based on university students exhibited healthier mental status and showed a lower sexual abuse prevalence estimate [3]. Finally, varying definitions of the term CSA may also influence the prevailing rates. For example, the age of consent to have sexual intercourse differs in cultural or legal contexts across countries, which may affect the conclusions from epidemiological studies and policy documents [59].

As for the symptoms-level relationship between smoking and PTSD of younger adults with CSA experience, four symptoms of PTSD (i.e. emotional cue reactivity, hypervigilance, nightmares, and difficulty concentrating) were both core and bridge symptoms among EC and CC users with CSA. In other words, these four symptoms might play critical roles in developing and perpetuating PTSD and smoking behaviours among the EC and CC groups. These results were consistent with previous studies. Previous literature has demonstrated that exposure to CSA could promote the risk of PTSD and smoking. A national longitudinal study among 15 197 adolescents reported that nicotine dependence and smoking were significantly related to CSA exposure [10]. Furthermore, the prevalence of cigarette smoking among people with PTSD was twice as high compared to the general population [60,61], which further illustrates the possibility of comorbidity between PTSD and smoking. In this study, “emotional cue reactivity” was a phenomenon in which emotions or memories evoked emotional and / or physiological responses. “Hypervigilance” refers to the experience of being in a state of high alert, constantly tense and “on guard” and always looking for real and presumed hidden dangers [62]. Several postulations below could potentially explain why emotional cue reactivity and hypervigilance were the most important symptoms that connected PTSD and smoking symptoms among CSA victims. First, hyperarousal symptoms (e.g. emotional cue reactivity, hypervigilance, and difficulty concentrating) have been suggested to play crucial roles in the maintenance of PTSD symptoms after a traumatic event and lead to mental disorders [63]. These symptoms could also contribute to an increased risk for smoking behaviour [64]. Second, the expectancy that smoking would reduce negative affect was found to mediate the association between the severity of PTSD and nicotine dependence [65]. Clinical research has shown that smoking increases neurohormones' levels to help modulate arousal, reduce emotional distress, and regulate mood [66]. Moreover, chronic smoking, in turn, was shown to promote inflammation which might increase the severity of PTSD [67]. Hence, the associations between PTSD and smoking symptoms were further strengthened, consistent with “emotional cue reactivity” and “hypervigilance” connecting PTSD and smoking among CSA victims.

Also, nightmares were the core and bridge symptom in the symptom dynamic between PTSD and smoking. Previous studies have reported more significant distress associated with nightmares among abuse victims during childhood and adolescence [68]. Furthermore, the nightmare prevalence is higher in college students with CSA experience than those without CSA [69]. Since nicotine results in dopamine release, which may relieve anxiety and induce relaxation [70], more patients with nightmares would like to smoke more for better sleep quality. Nonetheless, nicotine also equips with stimulant properties that are thought to be responsible for nightmares and other potential sleep problems associated with smoking [71]. Therefore, although smoking seems to relieve anxiety and calm down temporarily, nicotine stimulation increases the risk of nightmares during sleep. This could be why nightmares became an essential and bridge symptom between PTSD and smoking in the present study’s findings. Furthermore, since nicotine increases levels of the neurotransmitters, including glutamate and acetylcholine, in various regions of the brain, including the hippocampus (the memory centre) and the prefrontal cortex (the executive control centre), which are heavily involved in learning and concentration [72], smoking behaviours would more likely to result in concentration difficulties during work and learning.

Besides the shared four core and bridge symptoms in network structure, the difference in symptoms’ associations between EC and CC was also found. Results showed that there were significantly stronger associations between “nightmares” – “difficulty with restrictions” and “irritability / anger” – “more during wake up” among young EC users with CSA. The prevalence of EC use has risen dramatically among adolescents and young adults over the past decade globally [17,20]. Surrounded by aggressive marketing and social media influences, more young adults may generate interest in EC. Most of them may be attracted by the alluring tastes and various shapes and believe that EC exerts lower harm [21-24]. According to several studies conducted among the general population and patients with mental illness, most individuals believe that vapour from EC use is less harmful to others than smoke and second-hand smoke from CC use and that EC could help them reduce or quit smoking CC [73-75]. For example, a study among outpatients in substance use treatment programs reported that about half of the participants used EC to quit or reduce smoking, and 32% used EC for curiosity or experimentation [75]. In other studies, young adults used EC due to the availability of flavours such as mint, candy, fruit, and chocolate [76]. Furthermore, EC often has various trendy designs such as regular cigarettes, cigars, pipes, USB flash drives, pens, and other everyday items, which may attract more young adults to try EC. Considering these factors, more young adults could become interested in EC, especially for these participants with CSA experience, to calm down and relieve symptoms of PTSD. All these factors might strengthen the association of difficulty with restrictions. Besides, due to the convenient use and pleasant smell, which would not disturb other people, more young adults would use EC in the morning after waking up. With the effect of nicotine mentioned above, the connections between “irritability / anger” – “more during wake up” would also be more robust.

Although this was a large-scale study among young adults with CSA experience, several limitations should be considered. First, due to the cross-sectional design of this study, the causal relationship between these variables cannot be inferred. A longitudinal study should be conducted to clarify the direction of causal relationships between symptoms. Second, in this sample, not all participants met the clinical criteria for PTSD and / or smoking. Further research should compare the present findings with the network structure of comorbidity in a clinical sample of patients with comorbid PTSD and smoke. Third, assessing psychopathological variables relied on retrospective reports, which may cause recall bias. Finally, since the sample of this study was composed of young adults with CSA experience, findings of this study may not be generalized to general populations.

## CONCLUSIONS

This study found that four symptoms of PTSD (i.e. emotional cue reactivity, hypervigilance, nightmares, and difficulty concentrating) were central and bridge symptoms between PTSD and smoking behaviours among young EC and CC users with CSA experiences. Significant differences in symptom associations between EC and CC users also have been found. Therefore, more efforts should be made to restrict morning smoking among EC users exposed to CSA. In addition, target interventions and strategies among these core symptoms and associations should be implemented to relieve comorbid PTSD and smoking.

## Additional material


Online Supplementary Document

## References

[1] World Health Organization. Responding to children and adolescents who have been sexually abused: WHO clinical guidelines. 2017. 29630189

[2] Pan Y Lin XJ Liu JB Zhang SJ Zeng X Chen FL Prevalence of Childhood Sexual Abuse Among Women Using the Childhood Trauma Questionnaire: A Worldwide Meta-Analysis. Trauma Violence Abuse. 2021;22:1181-91. 10.1177/1524838020912867 32207395

[3] Moody G Cannings-John R Hood K Kemp A Robling M Establishing the international prevalence of self-reported child maltreatment: a systematic review by maltreatment type and gender. BMC Public Health. 2018;18:1164. 10.1186/s12889-018-6044-y 30305071 PMC6180456

[4] Batchelder AW Safren SA Coleman JN Boroughs MS Thiim A Ironson GH Indirect Effects From Childhood Sexual Abuse Severity to PTSD: The Role of Avoidance Coping. J Interpers Violence. 2021;36:NP5476-95. 10.1177/0886260518801030 30246600 PMC6785355

[5] Lindert J von Ehrenstein OS Grashow R Gal G Braehler E Weisskopf MG Sexual and physical abuse in childhood is associated with depression and anxiety over the life course: systematic review and meta-analysis. Int J Public Health. 2014;59:359-72. 10.1007/s00038-013-0519-5 24122075

[6] Ng QX Yong BZJ Ho CYX Lim DY Yeo WS Early life sexual abuse is associated with increased suicide attempts: An update meta-analysis. J Psychiatr Res. 2018;99:129-41. 10.1016/j.jpsychires.2018.02.001 29454220

[7] McTavish JR Sverdlichenko I MacMillan HL Wekerle C Child sexual abuse, disclosure and PTSD: A systematic and critical review. Child Abuse Negl. 2019;92:196-208. 10.1016/j.chiabu.2019.04.006 30999168

[8] McLean LM Toner B Jackson J Desrocher M Stuckless N The Relationship Between Childhood Sexual Abuse, Complex Post-Traumatic Stress Disorder and Alexithymia in Two Outpatient Samples: Examination of Women Treated in Community and Institutional Clinics. J Child Sex Abus. 2006;15:1-17. 10.1300/J070v15n03_01 16893816

[9] Campbell JA Walker RJ Egede LE Associations Between Adverse Childhood Experiences, High-Risk Behaviors, and Morbidity in Adulthood. Am J Prev Med. 2016;50:344-52. 10.1016/j.amepre.2015.07.022 26474668 PMC4762720

[10] Roberts ME Fuemmeler BF McClernon FJ Beckham JC Association Between Trauma Exposure and Smoking in a Population-Based Sample of Young Adults. J Adolesc Health. 2008;42:266-74. 10.1016/j.jadohealth.2007.08.029 18295135 PMC2675188

[11] Marshall EC Zvolensky MJ Vujanovic AA Gibson LE Gregor K Bernstein A Evaluation of smoking characteristics among community-recruited daily smokers with and without posttraumatic stress disorder and panic psychopathology. J Anxiety Disord. 2008;22:1214-26. 10.1016/j.janxdis.2008.01.003 18282685 PMC2600664

[12] Lee CJ Shpigel DM Segal KS Esan H Estey DR Hunt MG A review of research on smoking among United States Veterans with posttraumatic stress disorder (2006-2016). Mil Psychol. 2018;30:10-26. 10.1080/08995605.2017.1419020

[13] Trindade Fortes J Cano FG Miranda VA Kang HC Fontenelle LF Mendlowicz MV PTSD Predicts Smoking Cessation Failure in a Trauma-Exposed Population. J Dual Diagn. 2020;16:392-401. 10.1080/15504263.2020.1786615 32643580

[14] Kassel JD Stroud LR Paronis CA Smoking, stress, and negative affect: Correlation, causation, and context across stages of smoking. Psychol Bull. 2003;129:270-304. 10.1037/0033-2909.129.2.270 12696841

[15] Weaver TL Etzel JC Smoking patterns, symptoms of PTSD and depression: preliminary findings from a sample of severely battered women. Addict Behav. 2003;28:1665-79. 10.1016/j.addbeh.2003.08.041 14656552

[16] Hruska B Bernier J Kenner F Kenne DR Boros AP Richardson CJ Examining the relationships between posttraumatic stress disorder symptoms, positive smoking outcome expectancies, and cigarette smoking in people with substance use disorders: A multiple mediator model. Addict Behav. 2014;39:273-81. 10.1016/j.addbeh.2013.10.002 24144587

[17] Becker TD Arnold MK Ro V Martin L Rice TR Systematic Review of Electronic Cigarette Use (Vaping) and Mental Health Comorbidity Among Adolescents and Young Adults. Nicotine Tob Res. 2021;23:415-25. 10.1093/ntr/ntaa171 32905589

[18] Grana R Benowitz N Glantz SA E-cigarettes: a scientific review. Circulation. 2014;129:1972-86. 10.1161/CIRCULATIONAHA.114.007667 24821826 PMC4018182

[19] Gentzke AS Creamer M Cullen KA Ambrose BK Willis G Jamal A Vital Signs: Tobacco Product Use Among Middle and High School Students - United States, 2011-2018. MMWR Morb Mortal Wkly Rep. 2019;68:157-64. 10.15585/mmwr.mm6806e1 30763302 PMC6375658

[20] Hammond D Rynard VL Reid JL Changes in prevalence of vaping among youths in the United States, Canada, and England from 2017 to 2019. JAMA Pediatr. 2020;174:797-800. 10.1001/jamapediatrics.2020.0901 32364581 PMC7199169

[21] Mantey DS Cooper MR Clendennen SL Pasch KE Perry CL E-Cigarette Marketing Exposure Is Associated With E-Cigarette Use Among US Youth. J Adolesc Health. 2016;58:686-90. 10.1016/j.jadohealth.2016.03.003 27080732 PMC4900536

[22] Pepper JK Ribisl KM Brewer NT Adolescents’ interest in trying flavoured e-cigarettes. Tob Control. 2016;25 Suppl 2:ii62-6. 10.1136/tobaccocontrol-2016-053174 27633762 PMC5125087

[23] Peters RJ Jr Meshack A Lin MT Hill M Abughosh S The social norms and beliefs of teenage male electronic cigarette use. J Ethn Subst Abuse. 2013;12:300-7. 10.1080/15332640.2013.819310 24215223

[24] Brett EI Stevens EM Wagener TL Leavens ELS Morgan TL Cotton WD A content analysis of JUUL discussions on social media: Using Reddit to understand patterns and perceptions of JUUL use. Drug Alcohol Depend. 2019;194:358-62. 10.1016/j.drugalcdep.2018.10.014 30472576

[25] Fried EI van Borkulo CD Cramer AOJ Boschloo L Schoevers RA Borsboom D Mental disorders as networks of problems: a review of recent insights. Soc Psychiatry Psychiatr Epidemiol. 2017;52:1-10. 10.1007/s00127-016-1319-z 27921134 PMC5226976

[26] Borsboom D Cramer AOJ Network Analysis: an integrative approach to the structure of psychopathology. Annu Rev Clin Psychol. 2013;9:91-121. 10.1146/annurev-clinpsy-050212-185608 23537483

[27] Borsboom D A network theory of mental disorders. World Psychiatry. 2017;16:5-13. 10.1002/wps.20375 28127906 PMC5269502

[28] Beard C Millner AJ Forgeard MJ Fried EI Hsu KJ Treadway MT Network analysis of depression and anxiety symptom relationships in a psychiatric sample. Psychol Med. 2016;46:3359-69. 10.1017/S0033291716002300 27623748 PMC5430082

[29] Jones PJ Ma R McNally RJ Bridge Centrality: A Network Approach to Understanding Comorbidity. Multivariate Behav Res. 2021;56:353-67. 10.1080/00273171.2019.1614898 31179765

[30] McNally RJ Robinaugh DJ Wu GWY Wang L Deserno MK Borsboom D Mental Disorders as Causal Systems: A network approach to posttraumatic stress disorder. Clin Psychol Sci. 2015;3:836-49. 10.1177/2167702614553230

[31] Fried EI Eidhof MB Palic S Costantini G Huisman-van Dijk HM Bockting CLH Replicability and Generalizability of Posttraumatic Stress Disorder (PTSD) Networks: A Cross-Cultural Multisite Study of PTSD Symptoms in Four Trauma Patient Samples. Clin Psychol Sci. 2018;6:335-51. 10.1177/2167702617745092 29881651 PMC5974702

[32] von Elm E Altman DG Egger M Pocock SJ Gotzsche PC Vandenbroucke JP The Strengthening the Reporting of Observational Studies in Epidemiology (STROBE) Statement: guidelines for reporting observational studies. Int J Surg. 2014;12:1495-9. 10.1016/j.ijsu.2014.07.013 25046131

[33] Bernstein DP Stein JA Newcomb MD Walker E Pogge D Ahluvalia T Development and validation of a brief screening version of the Childhood Trauma Questionnaire. Child Abuse Negl. 2003;27:169-90. 10.1016/S0145-2134(02)00541-0 12615092

[34] Alexander N Kirschbaum C Wankerl M Stauch BJ Stalder T Steudte-Schmiedgen S Glucocorticoid receptor gene methylation moderates the association of childhood trauma and cortisol stress reactivity. Psychoneuroendocrinology. 2018;90:68-75. 10.1016/j.psyneuen.2018.01.020 29433075

[35] He J Zhong X Gao Y Xiong G Yao S Psychometric properties of the Chinese version of the Childhood Trauma Questionnaire-Short Form (CTQ-SF) among undergraduates and depressive patients. Child Abuse Negl. 2019;91:102-8. 10.1016/j.chiabu.2019.03.009 30856597

[36] Brewin CR Rose S Andrews B Green J Tata P McEvedy C Brief screening instrument for post-traumatic stress disorder. Br J Psychiatry. 2002;181:158-62. 10.1192/bjp.181.2.158 12151288

[37] Foa EB Riggs DS Dancu CV Rothbaum BO Reliability and validity of a brief instrument for assessing post-traumatic stress disorder. J Trauma Stress. 1993;6:459-73. 10.1002/jts.2490060405

[38] de Bont PA van den Berg DPG van der Vleugel BM de Roos C de Jongh A van der Gaag M Predictive validity of the Trauma Screening Questionnaire in detecting post-traumatic stress disorder in patients with psychotic disorders. Br J Psychiatry. 2015;206:408-16. 10.1192/bjp.bp.114.148486 25792693

[39] Knipscheer J Sleijpen M Frank L de Graaf R Kleber R ten Have M Prevalence of Potentially Traumatic Events, Other Life Events and Subsequent Reactions Indicative for Posttraumatic Stress Disorder in the Netherlands: A General Population Study Based on the Trauma Screening Questionnaire. Int J Environ Res Public Health. 2020;17:1725 10.3390/ijerph17051725 32155752 PMC7084195

[40] Walters JTR Blsson JI Shepherd JP Predicting post-traumatic stress disorder: validation of the Trauma Screening Questionnaire in victims of assault. Psychol Med. 2007;37:143-50. 10.1017/S0033291706008658 16959058

[41] Wu KK Leung PWL Wong CSM Yu PMW Luk BTC Cheng JPK The Hong Kong Survey on the Epidemiology of Trauma Exposure and Posttraumatic Stress Disorder. J Trauma Stress. 2019;32:664-76. 10.1002/jts.22430 31393657

[42] Heatherton TF Kozlowski LT Frecker RC Fagerstrom KO THE FAGERSTROM TEST FOR NICOTINE DEPENDENCE - A REVISION OF THE FAGERSTROM TOLERANCE QUESTIONNAIRE. Br J Addict. 1991;86:1119-27. 10.1111/j.1360-0443.1991.tb01879.x 1932883

[43] Becoña E Vazquez FL The Fagerstrom Test for Nicotine Dependence in a Spanish sample. Psychol Rep. 1998;83:1455-8. 10079737 10.2466/pr0.1998.83.3f.1455

[44] Payne TJ Smith PO McCracken LM McSherry WC Antony MM ASSESSING NICOTINE DEPENDENCE - A COMPARISON OF THE FAGERSTROM TOLERANCE QUESTIONNAIRE (FTQ) WITH THE FAGERSTROM TEST FOR NICOTINE DEPENDENCE (FTND) IN A CLINICAL-SAMPLE. Addict Behav. 1994;19:307-17. 10.1016/0306-4603(94)90032-9 7942248

[45] Huang CL Lin HH Wang HH The psychometric properties of the Chinese version of the Fagerstrom Test for Nicotine Dependence. Addict Behav. 2006;31:2324-7. 10.1016/j.addbeh.2006.02.024 16567055

[46] Yamada H Acton GS Tsoh JY Differential item functioning of the English and Chinese versions of the Fagerstrom Test for Nicotine Dependence. Addict Behav. 2009;34:125-33. 10.1016/j.addbeh.2008.09.003 18929444

[47] Kang H The prevention and handling of the missing data. Korean J Anesthesiol. 2013;64:402-6. 10.4097/kjae.2013.64.5.402 23741561 PMC3668100

[48] Epskamp S Borsboom D Fried EI Estimating psychological networks and their accuracy: A tutorial paper. Behav Res Methods. 2018;50:195-212. 10.3758/s13428-017-0862-1 28342071 PMC5809547

[49] Epskamp S Cramer AO Waldorp LJ Schmittmann VD Borsboom D qgraph: Network visualizations of relationships in psychometric data. J Stat Softw. 2012;48:1-18. 10.18637/jss.v048.i04

[50] van Borkulo CD Boschloo L Kossakowski J Tio P Schoevers RA Borsboom D Comparing network structures on three aspects: A permutation test. Psychol Methods. 2022. 10.1037/met0000476 35404628

[51] van Borkulo C, Epskamp S, Jones P, Haslbeck J, Millner A. Package ‘NetworkComparisonTest’. 2016

[52] Chernick MR. Bootstrap methods: A guide for practitioners and researchers: John Wiley & Sons; 2011.

[53] Costenbader E Valente TW The stability of centrality measures when networks are sampled. Soc Networks. 2003;25:283-307. 10.1016/S0378-8733(03)00012-1

[54] Epskamp S Fried EI A tutorial on regularized partial correlation networks. Psychol Methods. 2018;23:617. 10.1037/met0000167 29595293

[55] Ma Y Prevalence of Childhood Sexual Abuse in China: A Meta-Analysis. J Child Sex Abus. 2018;27:107-21. 10.1080/10538712.2018.1425944 29509078

[56] Tang K Qu X Li C Tan S Childhood sexual abuse, risky sexual behaviors and adverse reproductive health outcomes among Chinese college students. Child Abuse Negl. 2018;84:123-30. 10.1016/j.chiabu.2018.07.038 30086418

[57] Zhang R Liang Y Cao W Zeng L Tang K Sex and Urban&ndash;Rural Differences in the Relationship between Childhood Sexual Abuse and Mental Health among Chinese College Students. Int J Environ Res Public Health. 2022;19:9225. 10.3390/ijerph19159225 35954586 PMC9368484

[58] Finkelhor D Ji K Mikton C Dunne M Explaining lower rates of sexual abuse in China. Child Abuse Negl. 2013;37:852-60. 10.1016/j.chiabu.2013.07.006 23958110

[59] Mathews B Collin-Vezina D Child Sexual Abuse: Toward a Conceptual Model and Definition. Trauma Violence Abuse. 2019;20:131-48. 10.1177/1524838017738726 29333990 PMC6429628

[60] Budenz A Klein A Prutzman Y The Relationship Between Trauma Exposure and Adult Tobacco Use: Analysis of the National Epidemiologic Survey on Alcohol and Related Conditions (III). Nicotine Tob Res. 2021;23:1716-26. 10.1093/ntr/ntab057 33848342 PMC8562326

[61] Kearns NT Carl E Stein AT Vujanovic AA Zvolensky MJ Smits JA Posttraumatic stress disorder and cigarette smoking: A systematic review. Depress Anxiety. 2018;35:1056-72. 10.1002/da.22828 30192425

[62] Dalgleish T Moradi A Taghavi M Neshat-Doost H Yule W An experimental investigation of hypervigilance for threat in children and adolescents with post-traumatic stress disorder. Psychol Med. 2001;31:541-7. 10.1017/S0033291701003567 11305862

[63] Pérez LG Abrams MP López-Martínez AE Asmundson GJ Trauma exposure and health: The role of depressive and hyperarousal symptoms. J Trauma Stress. 2012;25:641-8. 10.1002/jts.21762 23184401

[64] Gabert-Quillen CA Selya A Delahanty DL Post-traumatic stress disorder symptoms mediate the relationship between trauma exposure and smoking status in college students. Stress Health. 2015;31:78-82. 10.1002/smi.2543 24424717

[65] Feldner MT Babson KA Zvolensky MJ Vujanovic AA Lewis SF Gibson LE Posttraumatic stress symptoms and smoking to reduce negative affect: An investigation of trauma-exposed daily smokers. Addict Behav. 2007;32:214-27. 10.1016/j.addbeh.2006.03.032 16644135

[66] Fu SS McFall M Saxon AJ Beckham JC Carmody TP Baker DG Post-traumatic stress disorder and smoking: a systematic review. Nicotine Tob Res. 2007;9:1071-84. 10.1080/14622200701488418 17978982

[67] Hosseinzadeh A Thompson PR Segal BH Urban CF Nicotine induces neutrophil extracellular traps. J Leukoc Biol. 2016;100:1105-12. 10.1189/jlb.3AB0815-379RR 27312847 PMC5069087

[68] Noll JG Trickett PK Susman EJ Putnam FW Sleep disturbances and childhood sexual abuse. J Pediatr Psychol. 2006;31:469-80. 10.1093/jpepsy/jsj040 15958722

[69] Agargun MY Kara H Ozer OA Selvi Y Kiran U Kiran S Nightmares and dissociative experiences: the key role of childhood traumatic events. Psychiatry Clin Neurosci. 2003;57:139-45. 10.1046/j.1440-1819.2003.01093.x 12667159

[70] Benowitz NL Pharmacology of nicotine: addiction, smoking-induced disease, and therapeutics. Annu Rev Pharmacol Toxicol. 2009;49:57-71. 10.1146/annurev.pharmtox.48.113006.094742 18834313 PMC2946180

[71] Jaehne A Loessl B Barkai Z Riemann D Hornyak M Effects of nicotine on sleep during consumption, withdrawal and replacement therapy. Sleep Med Rev. 2009;13:363-77. 10.1016/j.smrv.2008.12.003 19345124

[72] Valentine G Sofuoglu M Cognitive Effects of Nicotine: Recent Progress. Curr Neuropharmacol. 2018;16:403-14. 10.2174/1570159X15666171103152136 29110618 PMC6018192

[73] Cummins SE Zhu S-H Tedeschi GJ Gamst AC Myers MG Use of e-cigarettes by individuals with mental health conditions. Tob Control. 2014;23 suppl 3:iii48-53. 10.1136/tobaccocontrol-2013-051511 24824516 PMC4145659

[74] Hefner K Rosenheck R Merrel J Coffman M Valentine G Sofuoglu M E-cigarette use in VA service users with mental health and substance use disorders. J Dual Diagn. 2016;12:109-17. 10.1080/15504263.2016.1172895 27064443 PMC4976394

[75] Peters EN Harrell PT Hendricks PS O’grady KE Pickworth WB Vocci FJ Electronic cigarettes in adults in outpatient substance use treatment: Awareness, perceptions, use, and reasons for use. Am J Addict. 2015;24:233-9. 10.1111/ajad.12206 25809200

[76] Stein MD Caviness CM Grimone K Audet D Borges A Anderson BJ E-cigarette Knowledge, Attitudes, and Use in Opioid Dependent Smokers. J Subst Abuse Treat. 2015;52:73-7. 10.1016/j.jsat.2014.11.002 25483740 PMC4382432

