# Supplementary material for: A network approach to the symptom-level associations between smoking and posttraumatic stress disorder (PTSD) among young adults exposed to childhood sexual abuse
Source: J Glob Health. 2023 Jun 23;13:04037. doi: 10.7189/jogh.13.04037 (PMC10288921; doi:10.7189/jogh.13.04037)
Supplement: Online Supplementary Document [file jogh-13-04037-s001.pdf]

Supplementary materials

Figure S1. The correlations of symptoms between PTSD and FTND among two groups.

Figure S2. Stability of EI index by case dropping subset bootstrap among two groups.

Figure S3. Bootstrapped confidence intervals of edge weights among two groups.

Figure S4. Estimation of edge weight difference by bootstrapped difference test among two groups.

STable 1. The partial correlation coefficient between nodes of the network structure of PTSD and smoking problems among CC users exposed to CSA.

STable 2. The partial correlation coefficient between nodes of the network structure of PTSD and smoking problems among EC users exposed to CSA.

STable 3. The expected influence index value of each node among EC and CC users exposed to CSA.

STable 4. The value of significant difference on partial correlation coefficient of edges weights between EC and CC users exposed to CSA.

Figure S1. The correlations of symptoms between PTSD and FTND among two groups.

CC group EC group

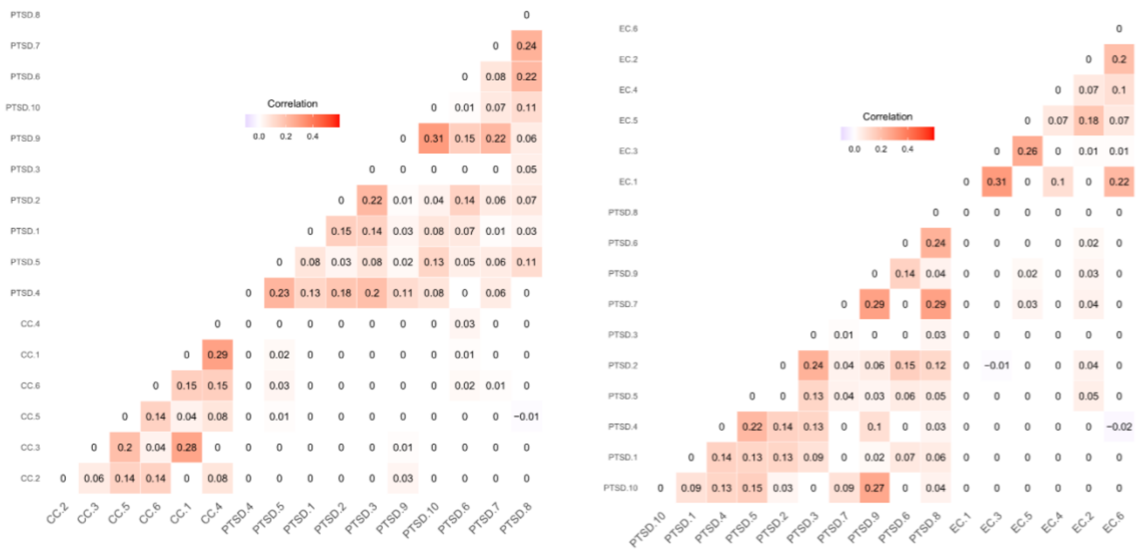

Figure S2. Stability of EI index by case dropping subset bootstrap among two groups.

CC group (CS-C=0.75)

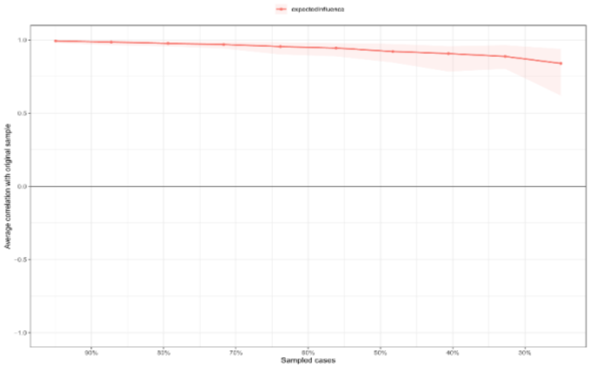

EC group (CS-C=0.67)

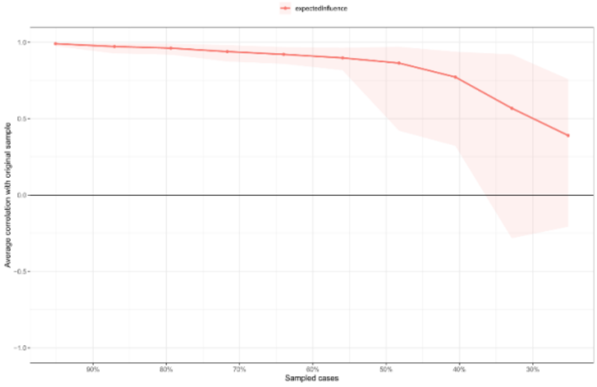

Figure S3. Bootstrapped confidence intervals of edge weights among two groups.

CC group

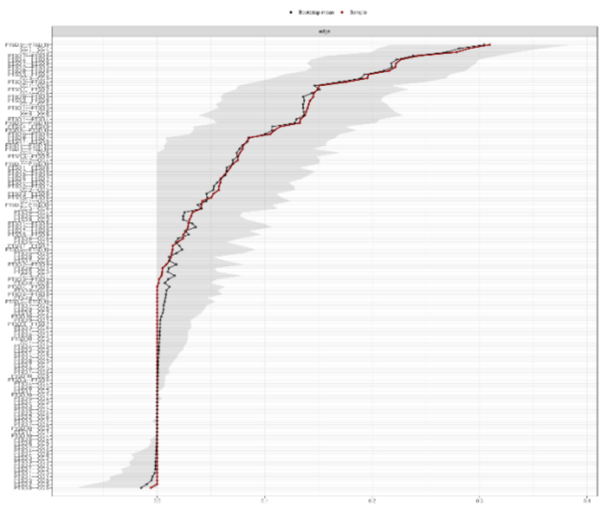

EC group

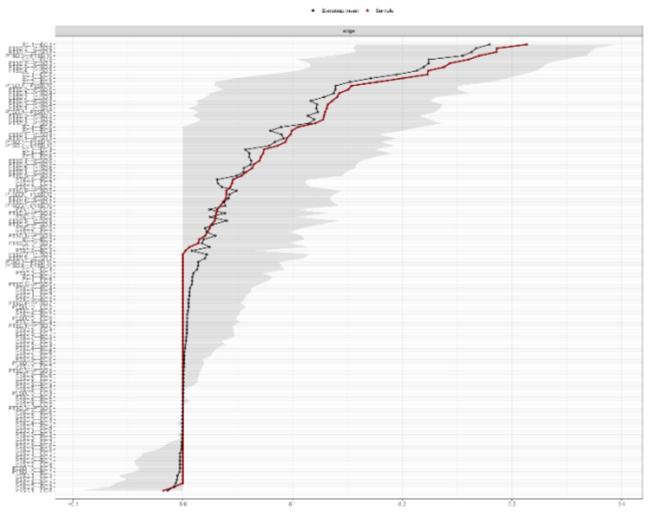

Figure S4. Estimation of edge weight difference by bootstrapped difference test among two groups.

CC group

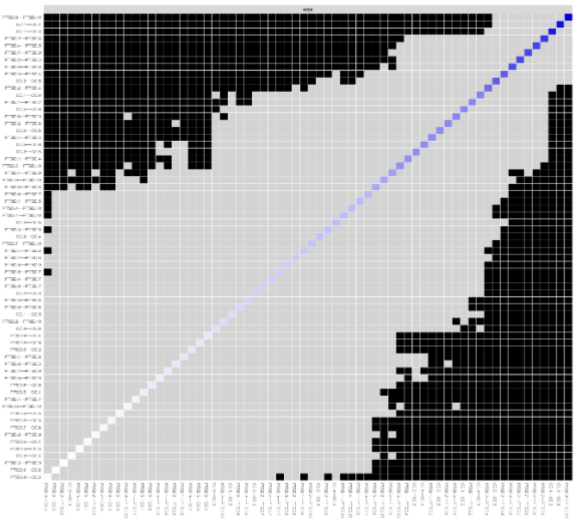

EC group

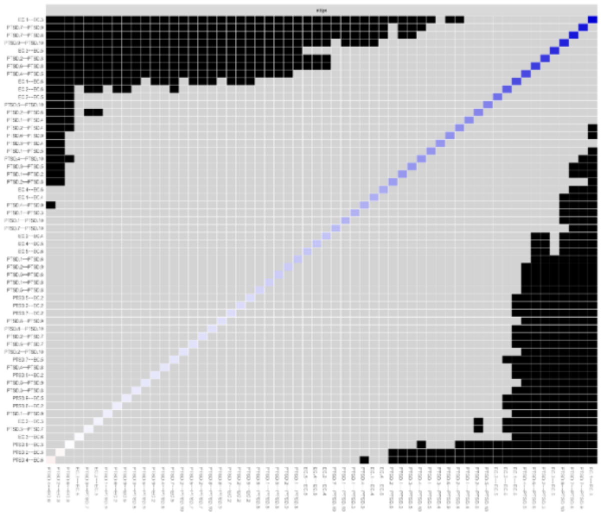

STable1. The partial correlation coefficient between nodes of the network structure of PTSD and smoking problems among CC users exposed to CSA.

	PTSD.1	PTSD.2	PTSD.3	PTSD.4	PTSD.5	PTSD.6	PTSD.7	PTSD.8	PTSD.9	PTSD.10	FTND.1	FTND.2	FTND.3	FTND.4	FTND.5	FTND.6
PTSD.1	0.00	0.15	0.14	0.13	0.08	0.07	0.01	0.03	0.03	0.08	0.00	0.00	0.00	0.00	0.00	0.00
PTSD.2	0.15	0.00	0.22	0.18	0.03	0.14	0.06	0.07	0.01	0.04	0.00	0.00	0.00	0.00	0.00	0.00
PTSD.3	0.14	0.22	0.00	0.20	0.08	0.00	0.00	0.05	0.00	0.00	0.00	0.00	0.00	0.00	0.00	0.00
PTSD.4	0.13	0.18	0.20	0.00	0.23	0.00	0.06	0.00	0.11	0.08	0.00	0.00	0.00	0.00	0.00	0.00
PTSD.5	0.08	0.03	0.08	0.23	0.00	0.05	0.06	0.11	0.02	0.13	0.02	0.00	0.00	0.00	0.01	0.03
PTSD.6	0.07	0.14	0.00	0.00	0.05	0.00	0.08	0.22	0.15	0.01	0.01	0.00	0.00	0.03	0.00	0.02
PTSD.7	0.01	0.06	0.00	0.06	0.06	0.08	0.00	0.24	0.22	0.07	0.00	0.00	0.00	0.00	0.00	0.01
PTSD.8	0.03	0.07	0.05	0.00	0.11	0.22	0.24	0.00	0.06	0.11	0.00	0.00	0.00	0.00	-0.01	0.00
PTSD.9	0.03	0.01	0.00	0.11	0.02	0.15	0.22	0.06	0.00	0.31	0.00	0.03	0.01	0.00	0.00	0.00
PTSD.10	0.08	0.04	0.00	0.08	0.13	0.01	0.07	0.11	0.31	0.00	0.00	0.00	0.00	0.00	0.00	0.00
FTND.1	0.00	0.00	0.00	0.00	0.02	0.01	0.00	0.00	0.00	0.00	0.00	0.00	0.28	0.29	0.04	0.15
FTND.2	0.00	0.00	0.00	0.00	0.00	0.00	0.00	0.00	0.03	0.00	0.00	0.00	0.06	0.08	0.14	0.14
FTND.3	0.00	0.00	0.00	0.00	0.00	0.00	0.00	0.00	0.01	0.00	0.28	0.06	0.00	0.00	0.20	0.04
FTND.4	0.00	0.00	0.00	0.00	0.00	0.03	0.00	0.00	0.00	0.00	0.29	0.08	0.00	0.00	0.08	0.15
FTND.5	0.00	0.00	0.00	0.00	0.01	0.00	0.00	-0.01	0.00	0.00	0.04	0.14	0.20	0.08	0.00	0.14
FTND.6	0.00	0.00	0.00	0.00	0.03	0.02	0.01	0.00	0.00	0.00	0.15	0.14	0.04	0.15	0.14	0.00

STable2. The partial correlation coefficient between nodes of the network structure of PTSD and smoking problems among EC users exposed to CSA.

	PTSD.1	PTSD.2	PTSD.3	PTSD.4	PTSD.5	PTSD.6	PTSD.7	PTSD.8	PTSD.9	PTSD.10	FTND.1	FTND.2	FTND.3	FTND.4	FTND.5	FTND.6
PTSD.1	0.00	0.13	0.09	0.14	0.13	0.07	0.00	0.06	0.02	0.09	0.00	0.00	0.00	0.00	0.00	0.00
PTSD.2	0.13	0.00	0.24	0.14	0.00	0.15	0.04	0.12	0.06	0.03	0.00	0.04	-0.01	0.00	0.00	0.00
PTSD.3	0.09	0.24	0.00	0.13	0.13	0.00	0.01	0.03	0.00	0.00	0.00	0.00	0.00	0.00	0.00	0.00
PTSD.4	0.14	0.14	0.13	0.00	0.22	0.00	0.00	0.03	0.10	0.13	0.00	0.00	0.00	0.00	0.00	-0.02
PTSD.5	0.13	0.00	0.13	0.22	0.00	0.06	0.04	0.05	0.03	0.15	0.00	0.05	0.00	0.00	0.00	0.00
PTSD.6	0.07	0.15	0.00	0.00	0.06	0.00	0.00	0.24	0.14	0.00	0.00	0.02	0.00	0.00	0.00	0.00
PTSD.7	0.00	0.04	0.01	0.00	0.04	0.00	0.00	0.29	0.29	0.09	0.00	0.04	0.00	0.00	0.03	0.00
PTSD.8	0.06	0.12	0.03	0.03	0.05	0.24	0.29	0.00	0.04	0.04	0.00	0.00	0.00	0.00	0.00	0.00
PTSD.9	0.02	0.06	0.00	0.10	0.03	0.14	0.29	0.04	0.00	0.27	0.00	0.03	0.00	0.00	0.02	0.00
PTSD.10	0.09	0.03	0.00	0.13	0.15	0.00	0.09	0.04	0.27	0.00	0.00	0.00	0.00	0.00	0.00	0.00
FTND.1	0.00	0.00	0.00	0.00	0.00	0.00	0.00	0.00	0.00	0.00	0.00	0.00	0.31	0.10	0.00	0.22
FTND.2	0.00	0.04	0.00	0.00	0.05	0.02	0.04	0.00	0.03	0.00	0.00	0.00	0.01	0.07	0.18	0.20
FTND.3	0.00	-0.01	0.00	0.00	0.00	0.00	0.00	0.00	0.00	0.00	0.31	0.01	0.00	0.00	0.26	0.01
FTND.4	0.00	0.00	0.00	0.00	0.00	0.00	0.00	0.00	0.00	0.00	0.10	0.07	0.00	0.00	0.07	0.10
FTND.5	0.00	0.00	0.00	0.00	0.00	0.00	0.03	0.00	0.02	0.00	0.00	0.18	0.26	0.07	0.00	0.07
FTND.6	0.00	0.00	0.00	-0.02	0.00	0.00	0.00	0.00	0.00	0.00	0.22	0.20	0.01	0.10	0.07	0.00

STable 3. The expected influence index value of each node among EC and CC users exposed to CSA.

Node	CC group	EC group
FTND.1	0.79	0.64
FTND.2	0.44	0.65
FTND.3	0.59	0.59
FTND.4	0.62	0.35
FTND.5	0.60	0.64
FTND.6	0.69	0.59
PTSD.1	0.72	0.74
PTSD.2	0.89	0.96
PTSD.3	0.69	0.64
PTSD.4	0.99	0.88
PTSD.5	0.86	0.86
PTSD.6	0.78	0.68
PTSD.7	0.82	0.82
PTSD.8	0.88	0.89
PTSD.9	0.96	1.00
PTSD.10	0.84	0.81

STable 4. The value of significant difference on partial correlation coefficient of edges weights between EC and CC users exposed to CSA.

	PTSD.2	PTSD.3	PTSD.4	PTSD.5	PTSD.6	PTSD.7	PTSD.8	PTSD.9	PTSD.10	FTND.1	FTND.2	FTND.3	FTND.4	FTND.5	FTND.6
PTSD.1	-	-	-	-	-	-	-	-	-	-	-	-	-	-	-
PTSD.2	-	-	-	-	-	-	-	-	-	-	-	-	-	-	-
PTSD.3	-	-	-	-	-	-	-	-	-	-	-	-	-	-	-
PTSD.4	-	-	-	-	-	-	-	-	-	-	-	-	-	-	-
PTSD.5	-	-	-	-	-	-	-	-	-	-	-	-	-	-	-
PTSD.6	-	-	-	-	-	-	-	-	-	-	-	-	-	-	-
PTSD.7	-	-	-	-	-	-	-	-	-	-	-	-	-	-	-
PTSD.8	-	-	-	-	-	-	-	-	-	-	-	-	-	-	-
PTSD.9	-	-	-	-	-	-	-	-	-	-	-	-	-	-	-
PTSD.10	-	-	-	-	-	-	-	-	-	-	-	-	-	-	-
FTND.1	-	-	-	-	-	-	-	-	-	-	-	-	-	-	-
FTND.2	**-0.04**	-	-	-	-	-	-	-	-	-	-	-	-	-	-
FTND.3	**0.01**	-	-	-	-	-	-	-	-	-	-	-	-	-	-
FTND.4	-	-	-	-	-	-	-	-	-	-	-	-	-	-	-
FTND.5	-	-	-	-	-	**-0.03**	-	-	-	-	-	-	-	-	-
FTND.6	-	-	**0.02**	-	-	-	-	-	-	-	-	-	-	-	-

* Only P values <0.05 of the edges with significant differences were presented in the table.
